# Hospital Administration

**Published:** 1899-06-03

**Authors:** 


### The Institutional Workshop.

### HOSPITAL ADMINISTRATION.

### THE LIVERPOOL HOSPITAL FOR CANCER AND SKIN DISEASES.

If ever we do feel a tinge of despair in regard to hospital affairs, it is when we see the management of a hospital drifting into the hands of individuals or of parties, when we see scandals hushed up by aid of so-called "inquiries" made in secret and therefore satisfactory to no one, and when we see dismissal served out as a reward to those who have shown sufficient independence to bring into public view and under public discussion abuses which they knew to exist. The Liverpool Hospital for Cancer and Skin Diseases seems to have been guilty of all these things, and is an admirable instance of the vices which an institution of this sort may develop in its management when it is enabled to withdraw itself from the controlling influence of public opinion. It cannot be said to be a very important institution, being in fact a small hospital of the so-called "special" class dealing with a curious mixture of diseases—cancer and skin diseases! One is reminded of the old-fashioned combination of eyes and throats, the only link between which would seem to lie in the fact that they are both investigated by aid of a lamp and mirror! Even this is a more substantial bond than any that can be discovered between skin and cancer. Putting on one side the suggestion of sham specialism which is offered by its name, it is not without interest to observe that it is again a special hospital of which complaint is made. When we come to consider what can be done to control such hospitals it is worth pointing out that, after all, their mismanagement is generally due to apathy on the part of the subscribers. So long as subscribers are to be found who are willing to put their hands in their pockets, but decline to take any further interest in the matter, we do not see how such evils are to be entirely stopped. If we are to accept the accounts of the affair given in the local press, accounts which have been commented on by *Truth* with all its accustomed vigour of expression, the ordinary governors allowed themselves to be outvoted by a number of persons who had qualified themselves as governors at the minimum amount only a few days beforehand, in order to vote at the meeting. Something of the same sort happened in London only a few weeks ago, again, of course, at a special hospital, the small number of ordinary governors who attended a meeting finding themselves in the hands of a sudden incursion of new men, and having to submit to a hostile vote on an important matter of policy. With proper rules, and with a moderate display of personal interest on the part of the ordinary subscribers, such things would be impossible, and we take it that the real cure for mismanagement in regard to hospitals is to be found, not in submitting their committees to outside control, and certainly not in government regulation, as in the very dangerous experiment now about to be tried in New York under the new Dispensaries Act, but rather in the development of that active personal interest in their management, on the part of men of substance and of business habits, which is the key to much of the success of our great London hospitals.

